# Growth Hormone: Therapeutic Possibilities—An Overview

**DOI:** 10.3390/ijms19072015

**Published:** 2018-07-11

**Authors:** Steve Harvey, Carlos G. Martinez-Moreno

**Affiliations:** 1 Department of Physiology, University of Alberta, Edmonton, AB T6G 2H7, Canada; 2 Departamento de Neurobiología Celular y Molecular, Instituto de Neurobiología, Campus Juriquilla, Universidad Nacional Autónoma de México, Querétaro, Qro. 76230, Mexico; cgmartin@comunidad.unam.mx

As a pituitary endocrine hormone, growth hormone (GH) has well established roles in promoting growth in individuals with GH deficiency [1]. It has also been used clinically to promote growth in states of short stature that lack a proven GH deficiency, such as in Turner’s syndrome [2,3], Prader-Willi syndrome [4], Noonan syndrome [5], idiopathic short stature [5], and chronic renal failure [6]; in conditions for which there is a short stature hometex (Shox) gene deficiency; and in children born small for gestational age (SGA) without catch-up growth [1]. It has also been used clinically in muscle wasting diseases such as HIV [7], and in catabolic diseases like cystic fibrosis and inflammatory bowel disease [8,9,10] and improving body composition [11]. Thus, although GH is synonymous with growth, it also has many other therapeutic actions, including novel roles in bone metabolism [12,13] and arthritis [14,15], rejuvenation [16,17,18] and longevity [19,20], neuro-rehabilitation [21,22] and neuro-function [23], inflammatory bowel disease [24,25], critical illness [26] for wound healing and burns [27,28,29], fibromyalgia [30], hypertension [31], and postmenopausal osteoporosis [32]. These newly defined roles for GH include actions around reparation of tissue damage independent of a state of GH-deficiency [22,25,28].

Exogenous GH is also used as an adjuvant therapy in in vitro fertilization and embryo transfer protocols (IVF-ET) for poor ovarian responders [33,34], and in experimental models of low sperm quality [35]. Exogenous GH has similarly been used to limit muscle atrophy in patients with peripheral nerve injury [36]. GH may also be a therapeutic treatment in Parkinson’s disease, in which the dopaminergic regulation of GH is abnormal [37]. Similarly, GH or IGF-I treatment has been proposed as a possible treatment for Alzheimer’s disease [38,39,40,41] and other neurodegenerative diseases [42].

In this issue, therapeutic considerations in the epigenetic regulation of gene expression in the GH-IGF-I axis and the essential role of GH and IGF-I in acetic acid-induced colitis are considered, together with manipulations of GH-IGF-I axis as a treatment strategy to reverse the effects of early life developmental programming. The role of autocrine human GH in cancer, and the role of GH in fat metabolism and in inflammatory bowel disease, its actions in liver function, and its promotion of distal innervation in children affected by caudal regression syndrome are also considered in this review. Actions of GH in the cardiovascular system, neuroregeneration and neuroprotection and their applications in adults with GH deficiency syndrome, and GHR mutations are also included in this special issue.
